# Supplementary material for: The Effects of Ultrasound Treatment of Graphite on the Reversibility of the (De)Intercalation of an Anion from Aqueous Electrolyte Solution
Source: Nanomaterials (Basel). 2022 Nov 8;12(22):3932. doi: 10.3390/nano12223932 (PMC9693535; doi:10.3390/nano12223932)
Supplement: Supplementary file 1 [file nanomaterials-12-03932-s001.zip › nanomaterials-1984743-supplementary.pdf]

Supplementary information

Article

The Effects of Ultrasound Treatment of Graphite on the Reversibility of the (De)Intercalation of an Anion from Aqueous Electrolyte Solution

Ghulam Abbas [1,2], Zahid Ali Zafar [2,3], Farjana J. Sonia [1], Karel Knížek [3], Jana Houdková [3], Petr Jiříček [3], Martin Kalbáč [1], Jiří Červenka [3] and Otakar Frank [1,*]

1 J. Heyrovsky Institute of Physical Chemistry, Czech Academy of Sciences, Dolejskova 2155/3, 183 23 Prague, Czech Republic
2 Department of Physical Chemistry and Macromolecular Chemistry, Faculty of Science, Charles University in Prague, Hlavova 2030, 128 43 Prague, Czech Republic
3 FZU—Institute of Physics of the Czech Academy of Sciences, Cukrovarnicka 10/112, 162 00 Prague, Czech Republic
* Correspondence: otakar.frank@jh-inst.cas.cz

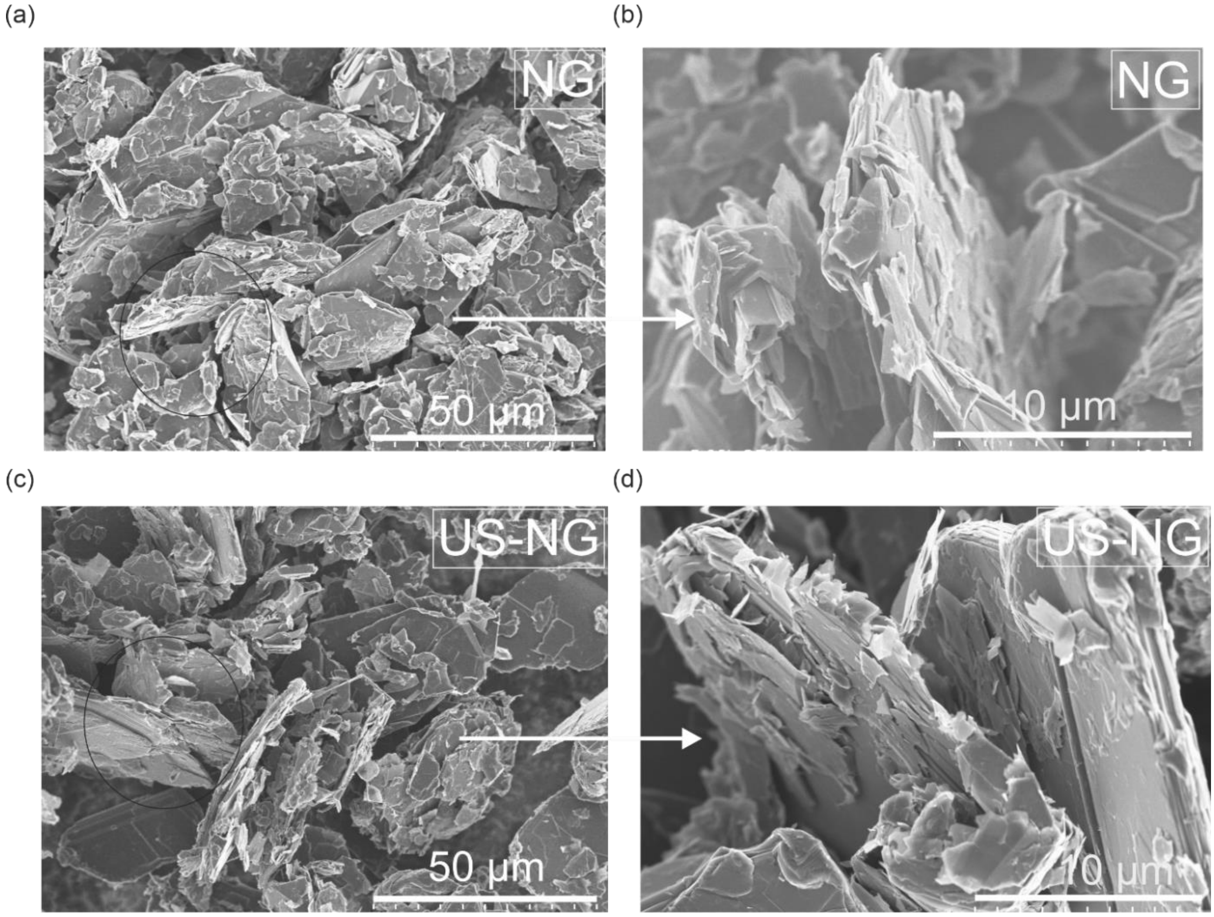

Figure S1. Characterization of NG and US-NG: (a) SEM image (50 μm scale) and (b) zoomed-in SEM image (10 μm) of NG; (c) SEM image and (d) zoomed-in SEM image of US-NG.

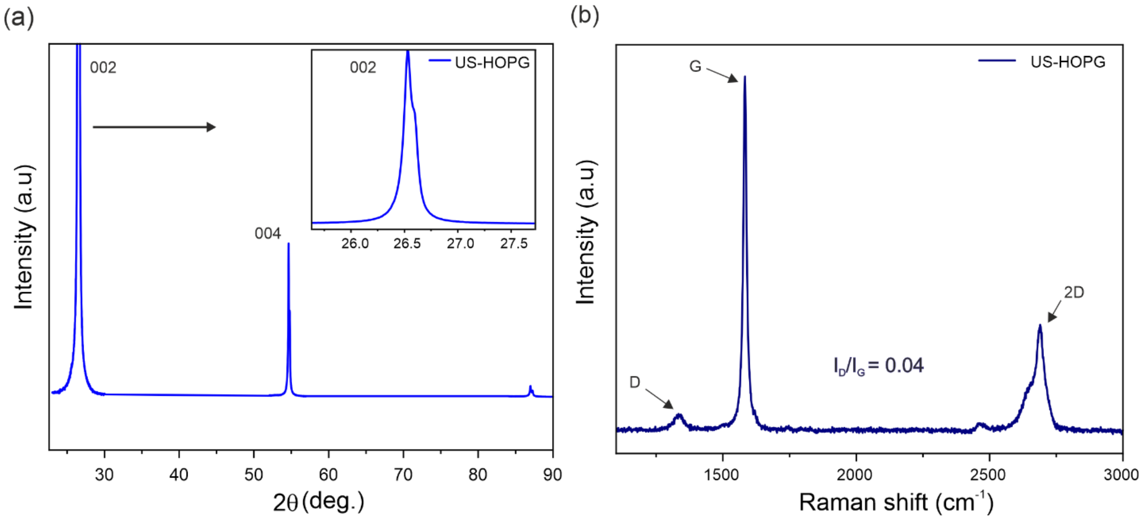

Figure S2. Characterization of US-HOPG: (a) XRD of US-HOPG (b) Raman spectrum of US-HOPG.

Table S1. Quantification of structural parameters of US-HOPG from XRD and Raman spectroscopy.

Material	g (%)	L_c (nm)	L_D (nm)	n_D (cm^{-2})	L_a (nm)
US-HOPG	96.66	97.50	84.16± 0.14	(4.48 ±0.25)×10^9	963.31

Calculation of the structural parameters for graphite

The structural parameters such as degree of graphitization (%) and crystallite domain size along the c-axis (Lc) for NG, US-NG, and US-HOPG were determined from XRD with accurate determination of 2θ, interlayer spacing (d_{002}) and width of the (002) XRD peak.

The degree of graphitization can be calculated by using the following equation [1].

$$g\ (\%) = \frac{0.3440 - d002}{0.34440 - 0.3354}\ x100 \tag{S1}$$

Where 0.3440 (nm) is the interlayer spacing of non-graphitized carbon and 0.3354 (nm) is the interlayer spacing of ideal graphite crystal, and d_{002} the interlayer spacing of the measured sample.

The crystallite domain size (Lc) along c-axis for the hexagonal graphitic structure can be calculated by using the Scherrer equation as follows [2,3]:

$$L = \frac{k\lambda}{\beta cos\theta} \tag{S2}$$

where k is the Scherrer constant and has value of 0.89 for c-axis, λ (nm) is the wavelength of incident X-rays, β (radian) is the full width at half maximum of the respective XRD peak, and θ is the observed Bragg's angle.

Raman spectroscopy has been widely used to probe the disorder in the crystal lattice of graphitic materials. The ratio of disorder-induced D peak in the lattice structure to the doubly degenerate Raman active G peak (I_D/I_G) enables us to calculate the lateral domain size along a-axis (La) by the following equation [4].

$$L_a(nm) = 2.4 \times 10^{-10} \lambda_L^4 \left(\frac{I_D}{I_G}\right)^{-1} \tag{S3}$$

where λ_L (nm) is the wavelength of the laser used to measure the Raman spectra.

The I_D/I_G ratio can also be used to estimate the inter-defect distance (L_D) and defect density (n_D) by the following equations as reported by Cancado *et al.* [5].

$$L_D^2\ (nm^2) = (1.8 \pm 0.5) \times 10^{-19} \lambda_L^4 \left(\frac{I_D}{I_G}\right)^{-1} \tag{S4}$$

$$nD\ ((cm^{-2})) = \frac{(1.8 \pm 0.5) \times 10^{22}}{\lambda_L^4} \left(\frac{I_D}{I_G}\right) \qquad \text{(S5)}$$

The electrochemical kinetics for (de)intercalation of ClO_4^- anion

The electrochemical kinetics for the (de)intercalation of the ClO_4^- anion can be revealed by the CV analysis. The ion diffusion or surface control process occurring during the intercalation/deintercalation of the ClO_4^- anion can be disentangled by using the power-law relationship between the peak current (i) and the scan rate (v) as analyzed from CV [6,7]. The power-law relationship can be written as:

$$i = a\,v^b \qquad \text{(S6)}$$

where a and b are adjustable parameters. The b value can be obtained from the slope of the *log(i) vs. log(v)* plot. The value of b = 0.5 indicates that the charge/discharge process is controlled by ion diffusion, while b = 1 indicates a purely surface-controlled process. The b value between 0.5 and 1.0 corresponds to the presence of both surface and diffusion-controlled processes.

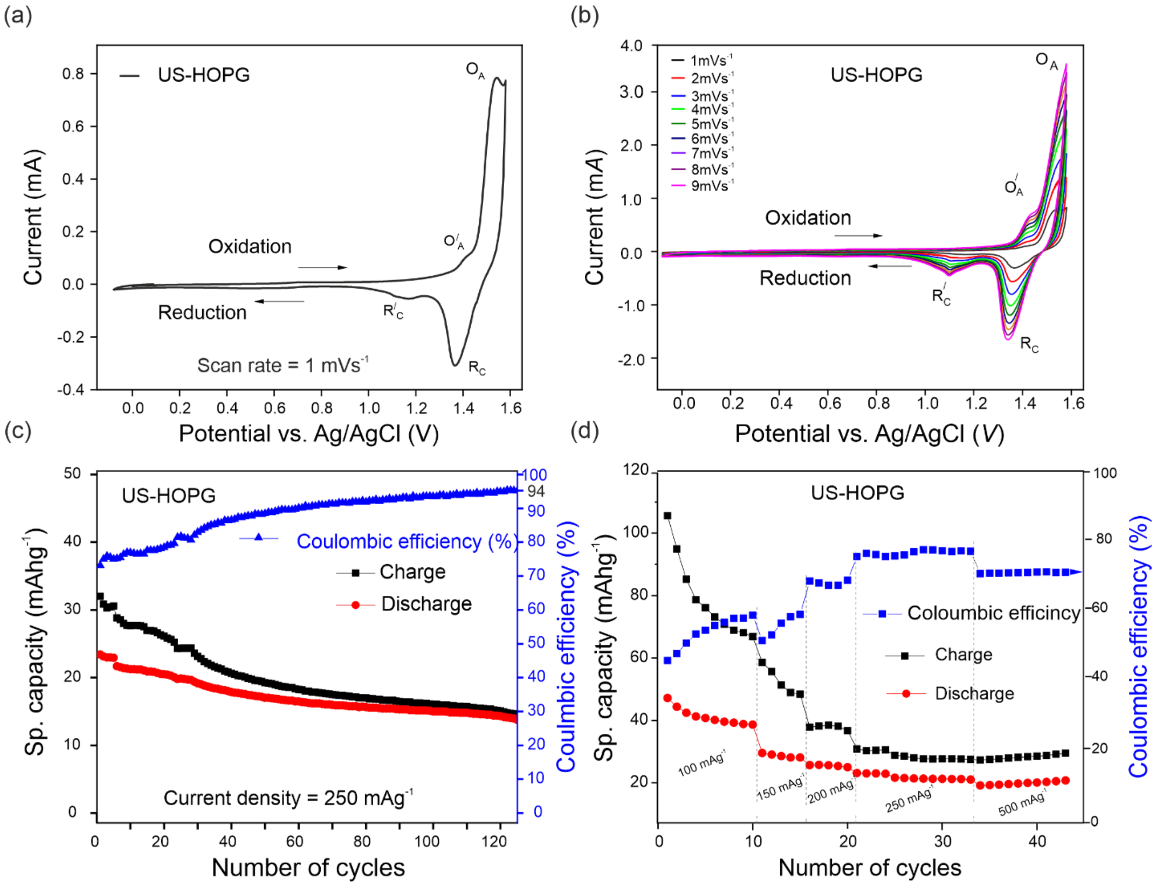

Figure S3. Electrochemical characteristics of US-HOPG using aqueous $Al(ClO_4)_3$ electrolyte solution: (a) cyclic voltammograms of US-HOPG (1 mVs^{-1}) (b) CV of US-HOPG at different scan rates (1 to 9 mVs^{-1}); (c) specific capacity of US-HOPG at a current density of 250 mAg^{-1}; (d) rate capability of US-HOPG at current densities from 100 mAg^{-1} to 500 mAg^{-1} using 2.4 M $Al(ClO_4)_3$ aqueous electrolyte solution.

Note 1:

The chemical composition of fresh US-NG material was analyzed by XPS (Figure S4). To elucidate the potential effects of airborn surface contamination, the sample was etched by Ar^+ ion clusters for 600 seconds (see Experimental section, main text, for more detail).

The presence of C, O, Fe, and Ar were observed in the wide-scan survey spectrum of US-NG (Figure S4a). The Fe signal originates from the sample holder and the presence of Ar could be due to the adsorbed Ar at the surface after sputtering. The concentration of Ar was zero before sputtering as can be seen from Table S2. Figure S4b shows the deconvoluted high resolution core level XPS spectrum of C1s. The shift in C=C and C-C peaks towards the lower frequency of 284.0 and 284.6 eV, respectively, could be attributed to the decoration of the graphite flakes by the functional groups. The designated oxygen-bearing groups attached to graphitic carbon as C-O, C=O, O-C-O and O-C=O, respectively, at binding energy (eV) of 285.5, 285.4, 288.4, and 289.4, and the hydroxyl groups C-OH and O=C-OH at binding energy (eV) of 286.4 and 290.3, respectively [8–11], can be seen in the high resolution deconvoluted core level XPS spectrum of C1s (Figure S4b). The presence of the carboxyl and hydroxyl functional groups at the graphite surface might originate from to the decomposition of the NMP solvent or surface oxidation as represented in Figure S4b. The presence of carboxylic and hydroxyl functional groups attached to graphitic carbon was also determined from corel level XPS spectrum of O1s (Figure S4c). The presence of C-O, C=O, O=C-O, C-OH, and COO/HOH at binding energy (eV) of 530.6, 531.6, 532.6 533.8 and 535.1 respectively [12–14], can be seen from the deconvoluted high resolution XPS spectrum of O1s.

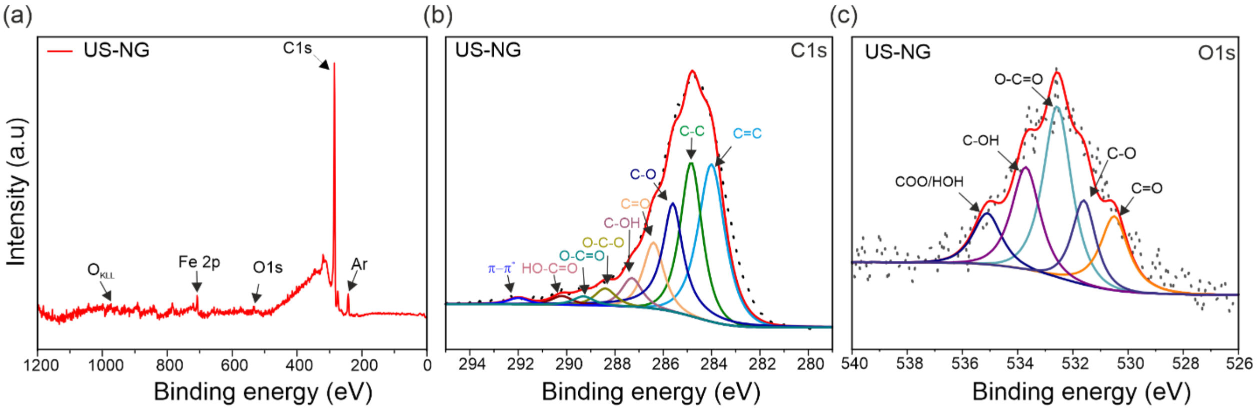

Figure S4. XPS analysis of fresh US-NG after Ar^{+} ion etching: (a) Wide-scan survey XPS spectrum; (b) the high-resolution deconvoluted XPS spectrum of the C1s core levels; (c) high-resolution core level XPS spectrum of O1s of US-NG.

Table S2. XPS elemental analysis of fresh US-NG material.

Sample	Etching time (sec)	C (at.%)	O(at.%)	Ar (at.%)
US-NG	0	96.8±0.09	3.2 ± 0.09	0.0
	600	94.7±0.10	1.0 ± 0.12	4.3±0.11

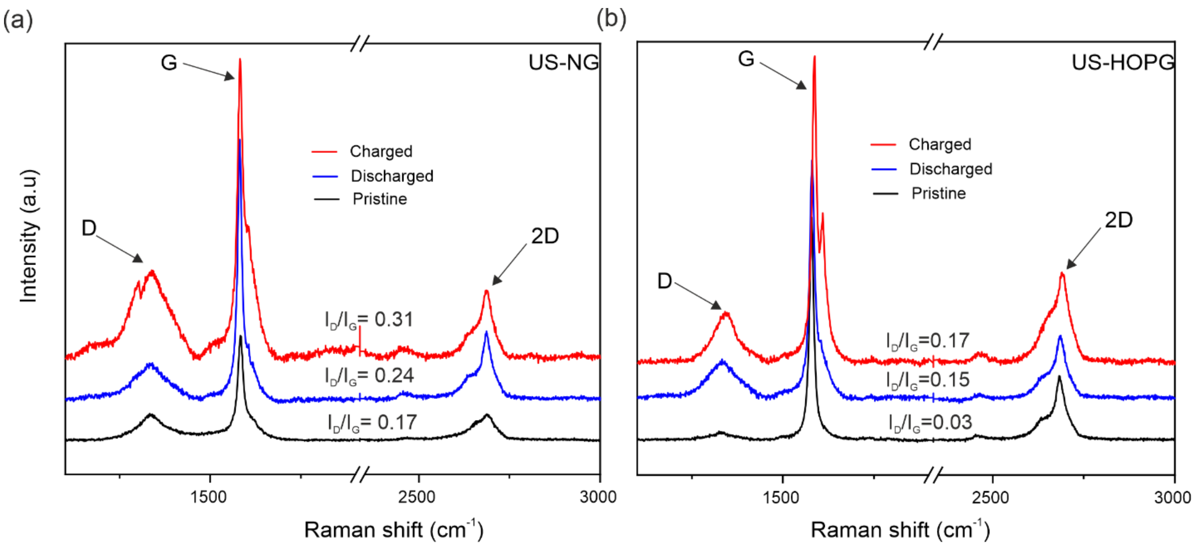

Figure S5. *Ex-situ* Raman spectra of graphite (a) *ex-situ* Raman spectra of US-NG and (b) US-HOPG during pristine, charged and discharged state in $Al(ClO_{4s})_3$ aqueous electrolyte solution.

Table S3. XPS elemental analysis of fresh, charged, and discharged US-NG.

Sample	Etching time (sec)	C (at.%)	Al (at.%)	Cl (at.%)	O (at.%)
Fresh	600	98.7 ± 0.39	---	----	1.3 ± 0.25
Charged	600	59.4 ± 0.68	7.5 ± 0.21	5.8 ± 0.12	27.3 ± 0.43
Discharged	600	91.3 ± 0.60	0.79 ± 0.17	1.0 ± 0.07	6.9 ± 0.32

References

1. Zou, L.; Huang, B.; Huang, Y.; Huang, Q.; Wang, C. An investigation of heterogeneity of the degree of graphitization in carbon–carbon composites. *Mater. Chem. Phys.* **2003**, *82*, 654–662. https://doi.org/10.1016/s0254-0584(03)00332-8.
2. Akikubo, K.; Kurahashi, T.; Kawaguchi, S.; Tachibana, M. Thermal expansion measurements of nano-graphite using high-temperature X-ray diffraction. *Carbon* **2020**, *169*, 307–311. https://doi.org/10.1016/j.carbon.2020.07.027.
3. Warren, B.E. X-Ray Diffraction in Random Layer Lattices. *Phys. Rev.* **1941**, *59*, 693–698. https://doi.org/10.1103/physrev.59.693.
4. Cançado, L.G.; Takai, K.; Enoki, T.; Endo, M.; Kim, Y.A.; Mizusaki, H.; Jorio, A.; Coelho, L.N.; Magalhães-Paniago, R.; Pimenta, M.A. General equation for the determination of the crystallite size La of nanographite by Raman spectroscopy. *Appl. Phys. Lett.* **2006**, *88*, 163106. https://doi.org/10.1063/1.2196057.
5. Cançado, L.G.; Jorio, A.; Ferreira, E.H.M.; Stavale, F.; Achete, C.A.; Capaz, R.B.; Moutinho, M.V.O.; Lombardo, A.; Kulmala, T.S.; Ferrari, A.C. Quantifying Defects in Graphene via Raman Spectroscopy at Different Excitation Energies. *Nano Lett.* **2011**, *11*, 3190–3196. https://doi.org/10.1021/nl201432g.
6. Sonia, F.J.; Jangid, M.K.; Ananthoju, B.; Aslam, M.; Johari, P.; Mukhopadhyay, A. Understanding the Li-storage in few layers graphene with respect to bulk graphite: Experimental, analytical and computational study. *J. Mater. Chem. A* **2017**, *5*, 8662–8679. https://doi.org/10.1039/c7ta01978e.
7. Sonia, F.J.; Jangid, M.K.; Aslam, M.; Johari, P.; Mukhopadhyay, A. Enhanced and Faster Potassium Storage in Graphene with Respect to Graphite: A Comparative Study with Lithium Storage. *ACS Nano* **2019**, *13*, 2190–2204. https://doi.org/10.1021/acsnano.8b08867.
8. Jayaramulu, K.; Dubal, D.; Nagar, B.; Ranc, V.; Tomanec, O.; Petr, M.; Datta, K.K.R.; Zboril, R.; Gómez-Romero, P.; Fischer, R.A. Ultrathin Hierarchical Porous Carbon Nanosheets for High-Performance Supercapacitors and Redox Electrolyte Energy Storage. *Adv. Mater.* **2018**, *30*, e1705789. https://doi.org/10.1002/adma.201705789.
9. Pei, S.; Cheng, H.M. The reduction of graphene oxide. *Carbon N. Y.* **2012**, *50*, 3210–3228.
10. Stobinski, L.; Lesiak, B.; Malolepszy, A.; Mazurkiewicz, M.; Mierzwa, B.; Zemek, J.; Jiricek, P.; Bieloshapka, I. Graphene oxide and reduced graphene oxide studied by the XRD, TEM and electron spectroscopy methods. *J. Electron Spectrosc. Relat. Phenom.* **2014**, *195*, 145–154. https://doi.org/10.1016/j.elspec.2014.07.003.
11. Yang, D.; Velamakanni, A.; Bozoklu, G.; Park, S.; Stoller, M.; Piner, R.D.; Stankovich, S.; Jung, I.; Field, D.A.; Ventrice, C.A., Jr.; et al. Chemical analysis of graphene oxide films after heat and chemical treatments by X-ray photoelectron and Micro-Raman spectroscopy. *Carbon N. Y.* **2009**, *47*, 145–152. https://doi.org/10.1016/j.carbon.2008.09.045.
12. Fan, L.-Z.; Liu, J.-L.; Ud-Din, R.; Yan, X.; Qu, X. The effect of reduction time on the surface functional groups and supercapacitive performance of graphene nanosheets. *Carbon* **2012**, *50*, 3724–3730. https://doi.org/10.1016/j.carbon.2012.03.046.
13. Yoshida, A.; Tanahashi, I.; Nishino, A. Effect of concentration of surface acidic functional groups on electric double-layer properties of activated carbon fibers. *Carbon* **1990**, *28*, 611–615. https://doi.org/10.1016/0008-6223(90)90062-4.

14. Bokare, A.; Nordlund, D.; Melendrez, C.; Robinson, R.; Keles, O.; Wolcott, A.; Erogbogbo, F. Surface functionality and formation mechanisms of carbon and graphene quantum dots. *Diam. Relat. Mater.* **2020**, *110*, 108101. https://doi.org/10.1016/j.diamond.2020.108101.
